# Experimental datasets on compliance level to minimum standard requirements among sandcrete block manufacturers in South Western Nigeria

**DOI:** 10.1016/j.dib.2018.08.058

**Published:** 2018-08-28

**Authors:** Adekunle M. Ajao, Kunle E. Ogundipe, Babatunde F. Ogunbayo, Obinna D. Nduka

**Affiliations:** Department of Building Technology, Covenant University, Ota, Nigeria

**Keywords:** Aggregates, Experimental procedure, Sandcrete blocks, Standards, Strength parameters

## Abstract

The data in this article are related to “Assessment of Sandcrete Blocks Manufacturers ‘Compliance to Minimum Standard Requirements by Standard Organisation of Nigeria in Southwest, Nigeria” (Ajao et al., 2018). The data shows the compliance level of Sandcrete Block Manufacturers to Minimum Standard Requirement in Southwest, Nigeria. Experimental procedures carried out on aggregates and sandcrete blocks included sieve analysis to determine grading distribution sizes and bulk density and compressive strength to determine the strength parameters. The results of the experiment were presented in charts and graphs.

**Specification Table**

**Table:** 

Subject area	Building Construction, Building Science
More specific subject area	Building materials Development
Type of data	Charts and graphs
How data was acquired	Experimental procedures in the laboratory and simple statistical tools were employed for the analyses
Data format	Raw data obtained from experimental procedures were processed.
Experimental factors	Several tests on properties and strength parameters of aggregate samples and sandcrete hollow blocks such as Bulk Density, Sieve Analysis and compressive strength were carried out.
Experimental features	Engineering properties of sandcrete blocks and laboratory tests.
Data source location	Samples of fine aggregates and 54 sandcrete blocks comprising 225 mm and 150 mm were gotten from block production sites within three states; Oyo, Ondo, and Lagos State. Nigeria. The control sample was gotten in Ota, Ogun State, Nigeria.
Data accessibility	The data are available with this article.
Related research article	Ajao, A. M., Ogunbayo, B. F., Ogundipe, K. E., Bamigboye, G., Ogunde, A., & Tunji-Olayeni, P. F. (2018). Assessment of Sandcrete Blocks Manufacturers ‘Compliance to Minimum Standard Requirements by Standard Organisation of Nigeria in Southwest, Nigeria. *International Journal of Applied Engineering Research*, *13*(6), 4162–4172.

**Value of the data**

• The data provided detailed experimental procedures on how sandcrete blocks manufacturers’ complied with minimum standard requirements.
• The data provided the best method for regulating the standards of sandcrete blocksproduction.
• The data can be used in any research that involved studying of sandcrete blocksproduction process and strength evaluation.
• The data is detailed and it can be adopted in construction industries and Built-environment professionals for policy making.

## 1 Data

**Fig. 1:**
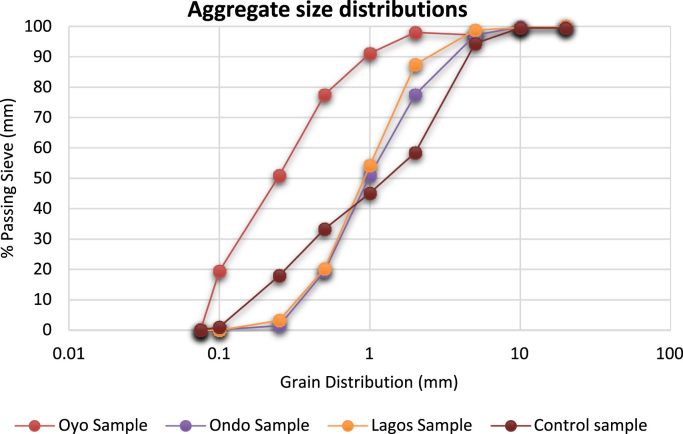
Particle size distribution for Oyo State, Ondo State, Lagos State and Control Sample.

**Fig. 2:**
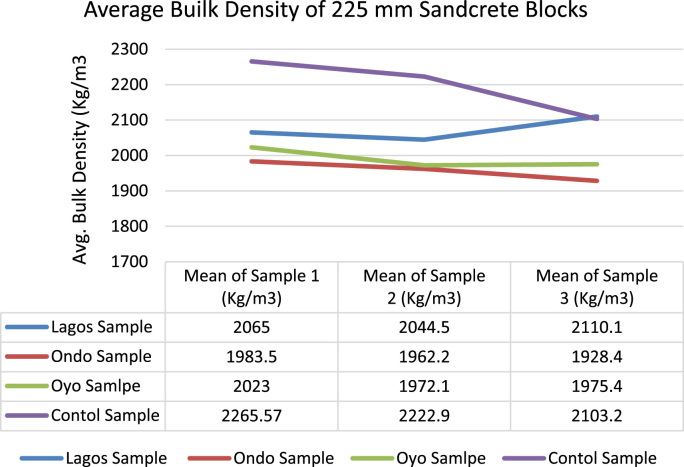
Mean Bulk Density kg/m^3^ of 225 mm sandcrete blocks.

**Fig. 3:**
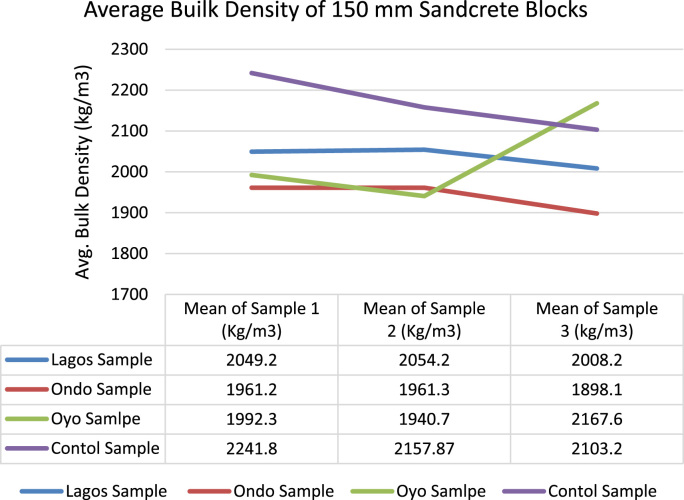
Mean Bulk Density kg/m^3^ of 150 mm sandcrete blocks.

The data of this article examined compliance level to minimum standard requirements among sandcrete block manufacturers in South Western Nigeria [1], [2], [3], [4], [5], [6], [7], [8], [9], [10], [11], [12], [13], [14], [15], [16], [17], [18]. The data presented in Fig. 1 were gotten from the analyses of particle size distribution of fine aggregates obtained from the sandcrete blocks manufacturers within three States and the control sample. The result of samples gotten from South West along with laboratory sample conformed to the overall grading and coarse grading limit according to [6]. The particle distribution of the samples showed that they were suitable for sandcrete block moulding [19]. Data of Bulk Density of sandcrete blocks 225 mm and 150 mm are shown in Fig. 2, Fig. 3 which represented South West, Nigeria and were in line with the recommended value of minimum limit of 1920 kg/m^3^ for individual blocks and 2020 kg/m^3^ for an average of three or more blocks [4], [8], [11].

## 2 Experimental design, materials and methods

The sample of fine aggregates and 54 sandcrete blocks comprising 225 mm and 150 mm used for this data were obtained from blocks production sites in these three states; Oyo, Ondo, and Lagos State. These three States were chosen within six States in South West to avoid similarity of result from all states. And Samples collected from these States were tested with necessary apparatus and parameters such as: Sieve analysis, Bulk Density, and compressive strength test. For better comparison of the test results with laid down standards from Standard Organisation of Nigerian, 18 numbers of controlled experimental units which comprised 225 mm and 150 mm were also produced for usual tests in laboratory, drinkable water used for the controlled experiment conformed to [20] while Ordinary Portland cement (OPC) grade 42.5 N used was in good condition. The following tests; Sieve analysis, Bulk Density, and compressive strength were also performed. To provide fair treatment for comparison and analysis of the results of sandcrete blocks obtained from the sandcrete blocks suppliers and controlled experimental units of mix design (1:8,) cement: sand, which were produced under the same atmospheric condition and employing the same production process as the suppliers. These results were then compared with the minimum standards. The data presentation is similar to that of [1], [21], while safety procedures recommended in [22], [23], [24] were strictly followed in preparing the control samples.

**Fig. 4:**
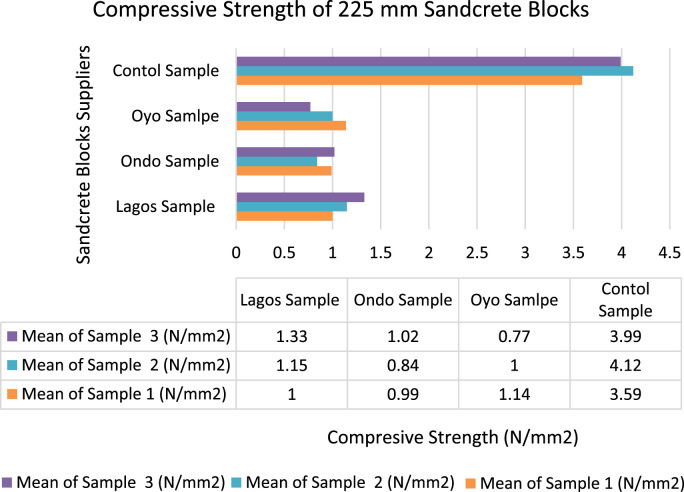
Compressive strength of 225 mm sandcrete blocks.

**Fig. 5:**
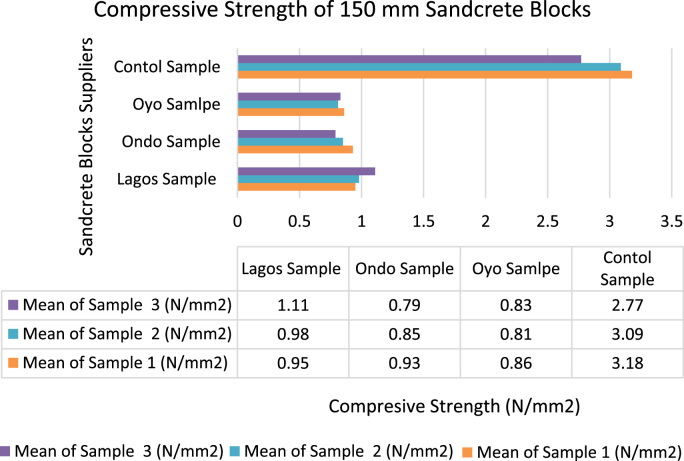
Compressive strength of 150 mm sandcrete blocks.

Fig. 4, Fig. 5 indicated the varieties of strength parameters of compressive strength test observed from the study. The results of the mean compressive strength of the sandcrete blocks (150 mm and 225 mm) of the three States were not in conformity with minimum standards required [8] except the controlled masonry units produced in the Laboratory. This showed that most of sandcrete blocks sold to the general public in South West were of low standard. The low standard of sandcrete blocks was as a result of poor mix-design, inadequate production process, insufficient curing period [11], [12], [13], [14] and lack of governmental agency and professional control [1]. The data presented lasting method of ameliorating the ugly practice among the manufacturers by involving regulatory bodies to enforce the use of registered stamp on their sandcrete block in each State.
